# Eco-epidemiological study of seropositivity against Rickettsia and Leptospira agents in rural areas of Urabá, Colombia

**DOI:** 10.21203/rs.3.rs-3760267/v1

**Published:** 2024-01-04

**Authors:** Mariana Torres-Bustamante, Omar Cantillo-Barraza, Albert I. Ko, Elsio A. Wunder, Juan C. Quintero-Vélez

**Affiliations:** Universidad de Antioquia; Universidad de Antioquia; Yale School of Public Health; Yale School of Public Health; Universidad de Antioquia

**Keywords:** concomitant exposure, multinomial model, Leptospira, Rickettsia, factor associated, seroconversion, seroincidence, seroprevalence

## Abstract

Rickettsioses and leptospirosis are infectious diseases that are often underdiagnosed due to a lack of knowledge about their epidemiology, pathophysiology, diagnosis, management, among others.

**Objetive:**

to characterize the seroprevalence and seroincidence of both *Rickettsia* and *Leptospira* agents and determine the risk factors for these outcomes in rural areas of Urabá, Antioquia.

**Methods:**

a secondary data analysis using information on *Rickettsia* and *Leptospira* exposure from a prior prospective study that explored sociocultural and ecological aspects of *Rickettsia* infection in rural Urabá, Colombia. A multinomial mixed logistic regression model was employed to analyze factors linked to seroprevalent cases of *Rickettsia*, *Leptospira* and both, along with descriptive analyses of seroincident cases.

**Results:**

the concomitant seroprevalence against *Rickettsia*and *Leptospira* was 9.38% [95%CI 6.08%–13.37%] (56/597). The factors associated with this seroprevalence were age (ORa= 1.02 [95%CI 1.007–1.03]), male gender (ORa= 3.06 [95%CI 1.75–5.37]), fever history (ORa= 1.71 [95%CI 1.06–2.77]) the presence of breeding pigs (ORa= 2.29 [95%CI 1.36–3.88]), peridomicile yucca crops(ORa= 2.5 [95%CI 1.1–5.62]), and deforestation practices(ORa= 1.74 [95%CI 1.06–2.87]). The concomitant seroincidence against *Rickettsia* and *Leptospira* was 1.09% (3/274) [95%CI 0.29%–4.05%], three cases were female, with a median age of 31.83 years-old (IQR 8.69–56.99). At the household level, all the seroincident cases had households built partially or totally with soil floors, wooden walls, and zinc roofs. Two seroincident cases described the presence of equines, canines, and domestic chickens in intra or peri-domicile. Finally, two cases were exposed to synanthropic rodents, and one case to tick infestation.

**Conclusion:**

there is evidence of seroprevalent and seroincident cases of seropositivity against both *Rickettsia* and *Leptospira* in rural areas of Urabá, Colombia. These findings can help improve public health surveillance systems in preventing, detecting, and attending to the different clinical cases caused by these pathogens.

## Introduction

Rickettsiae and leptospires are microorganisms transmitted by ectoparasites and environmental sources contaminated with the urine of infected mammals, respectively (1). The severity of the illness generated by these microorganisms depends on the virulence of bacteria species responsible for the infection (2–5). The transmission of these agents to humans is facilitated by the increased interaction between the vectors, amplifier mammals, and humans largely due to deforestation, climatic change, the complex infection cycles of these microorganism that involve several amplifier hosts (synanthropic rodents, opossums, canines, and pigs) in common, and socio-cultural factors, such as attitudes and practices, as well as the marginalization and poverty of the communities affected by these health events (6–10).

Antioquia, a region of Colombia located in the northwest of the country, exhibits several conditions that heighten the probability of human infection by various infectious agents, including *Rickettsia* and *Leptospira*. Conditions such as low quality of life index, substandard housing, precarious access to public services and education, widespread poverty, and barriers to healthcare access contribute to this heightened risk, alongside ongoing deforestation and agricultural expansion in the region (11).

Previous evidence has demonstrated the transmission of spotted fever group rickettsiae (SFG) and leptospires in rural areas of Urabá, Antioquia, with documented cases of exposure, infection, and disease. In this area, a seroprevalence of 25.6% (95% CI 22.11% – 29.12%) against rickettsiae SFG and a seroincidence of 6.23% (95%CI: 3.67–9.68%) were estimated (12, 13). Similarly, a seroprevalence against *Leptospira* of 27.81% (95% CI: 24.78% – 30.84%) and a seroincidence of 14.60% (95% CI: 11.20% – 17.13%) have been recorded (14).

While Colombia has had a leptospirosis surveillance system in place since 2007, rickettsiosis events are not currently classified as mandatory notification events and are instead grouped within unspecified fevers in hospital surveillance (15, 16). From a public health perspective, these health events are of significant concern due to their high potential to lead to disease outbreaks with high lethality and a substantial likelihood of co-infection cases. In Urabá, cases of potential co-infection of rickettsiosis-leptospirosis, including co-infections with other parasitic and viral agents, have been documented (17–19).

The evidence of cases of exposure, infection, and disease caused by rickettsiae SFG and leptospires in rural areas of Urabá suggest the possibility of concomitant exposure or co-infection cases by these microorganisms. Such occurrences may be influenced by sociocultural or ecological factors that increase the likelihood of exposure to both microorganisms, potentially leading to more severe illnesses in susceptible individuals. Therefore, this study aims to characterize eco-epidemiologically the seroprevalence and seroincidence to both *Rickettsia* and *Leptospira* in rural areas of Urabá, Antioquia. Likewise, the hypothesis of this study was that socio-demographic and ecologic conditions exist that favor the presentation of potential concomitant seropositivity to *Rickettsia* and *Leptospira*.

## Materials and methods

### Location and study design

**Figure 1:**
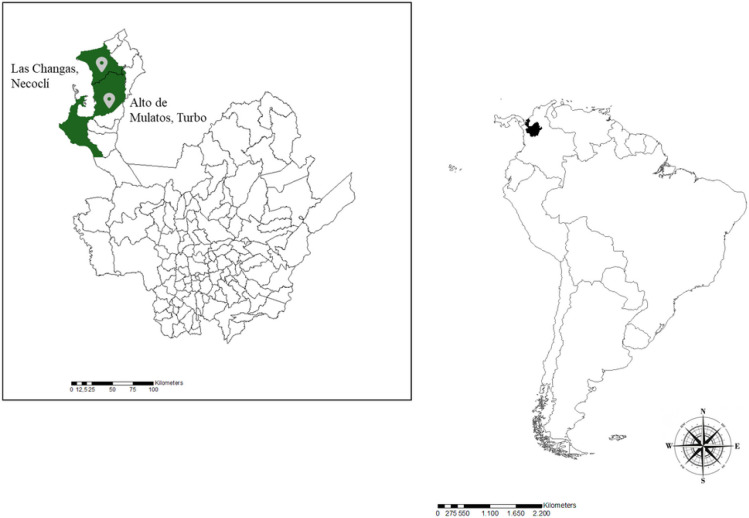
Map the of study site: Turbo and Necoclí municipalities, Antioquia, Colombia.

Using predefined inclusion and exclusion criteria, we designed a secondary data analysis using data collected from a prospective study that assess *Rickettsia* SFG and *Leptospira* infections in nine hamlets located in the rural areas of the Urabá region (12–14). These hamlets comprised five in Alto de Mulatos, Turbo, Colombia (8° 08’ 12.5” N 76° 33’ 01.7” W) and four in Las Changas, Necoclí, Colombia (8° 32’ 52.5” N 76° 34’ 23.7” W) (Fig. 1). The selection of these hamlets for the baseline time (T0) (conducted between November 2015 and January 2016) was based on ecological factors related to the transmission of infectious tropical diseases, including the presence of wild mammals, mosquitoes, and ticks, as well as sociodemographic characteristics such as the number of households, the absence of illegal armed groups present in the area, access routes, and short distances between rural and urban centers (< 2 hours travel time by horse) to facilitate study logistics. Follow-up data collection was performed one year later (T12) (between November 2016 and January 2017).

### Sampling design

A census tract was conducted to obtain a sample frame, finding a total of 461 households inhabited by 1915 persons. A cluster random sample was designed with households as the sample unit and the inhabitants as the analysis unit. A sample size of 208 households inhabited by 865 persons was calculated, considering a 95% level of confidence, 5% error, and 41% expected seroprevalence (20).

### Serologic testing

An indirect immunofluorescence assay (IFA) was used to detect the IgG against SFG rickettsiae. A seroprevalent case was defined as a human with an IgG titer ≥ 128 at T0 (21). A seroincident case was defined as a serological conversion of IgG titers (negative at baseline study to a seropositive titer of ≥ 128 at the follow-up study) or at least a four-fold increase in the IgG endpoint titer at follow-up T12. The IFA *R. rickettsii, R. amblyommatis, R. parkeri*, and *R. belli* to detect the potential exposure of these rickettsial species (12).

Seropositivity against *Leptospira* was analyzed through microscopic agglutination testing (MAT). A panel of 32 *Leptospira* strains of different species and serogroups was used to select the final panel to evaluate all serum samples, as described previously. A total of 16 antigens were selected to analyze all the participants’ samples (**S1 Table**) (14). The serologic evaluation was conducted using a dilution of 1:50 and 1:100. A seroprevalent case was determined when 50% or more leptospires for at least one serogroup strain were agglutinated at ≥ 1:50 titer. All seropositive samples were titrated to determine the higher titer for each strain. Seroincident cases of *Leptospira* infection were determined by seroconversion from seronegative to seropositive or by a minimum fourfold increase in titers between baseline and follow-up samples. The presumptive infecting serogroup in both seroprevalent and seroincident cases was determined as the serogroup with the highest antibody titer.

### Outcomes

In the baseline measure T0, a multinomial outcome was considered as i) seronegative cases, ii) seroprevalent case against *Rickettsia*, iii) seroprevalent cases against *Leptospira*, and iv) seroprevalent concomitant cases (defined as serum sample seropositivity against both agents *Rickettsia* and *Leptospira*). At follow-up T12, the multinomial outcomes included i) seronegative cases, ii) seroincident cases of *Rickettsia*, iii) seroincident cases of *Leptospira*, iv) seroincident cases against both *Rickettsia* and *Leptospira* ( seroincident concomitant cases).

**Figure 2:**
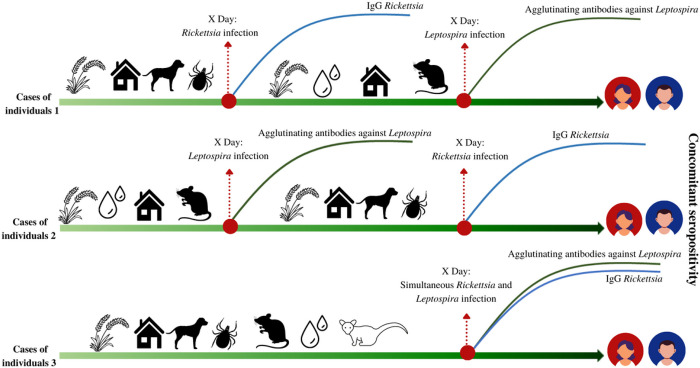
Scenarios for concomitant seropositivity against *Rickettsia* and *Leptospira*

The concomitant seropositivity to both microorganisms could occur in three scenarios: simultaneous exposure to *Rickettsia* and *Leptospira*, exposure to *Rickettsia*, and later to *Leptospira*, or vice versa (Fig. 2).

Cases of individuals 1: primary infection with *Rickettsia* with secondary antibodies response, later *Leptospira* infection with secondary antibodies response.

Cases of individuals 2: primary infection with *Leptospira* with secondary antibodies response, later *Rickettsia* infection with secondary antibodies response.

Cases of individuals 3: Simultaneous infection with *Leptospira* and *Rickettsia* with antibodies response against both microorganisms.

### Principal exposure variable, co-variables, and data sources

Data on individual, household, and hamlet variables were collected through an epidemiological questionnaire, as previously described (12, 13). The main exposure variable was working outdoors (farmers, ranchers, day laborers, soldiers, and agricultural workers, among others). This variable was analyzed at T0 in terms of previous occupation (within the last five years) and current occupation (at the time of application of the questionnaire). For T12 the occupation was evaluated in the previous 12 months. In addition, the individual covariables, such as age in years, gender, ethnicity, time of residence in the area, history of fever, and previous tick infestation, were analyzed.

Household co-variables evaluated included household location (urban or rural), proximity among households (widely dispersed, dispersed, concentrated, and very concentrated), building material (total or partial) of floors, walls, and roof, garbage disposal area, sewerage, and aqueduct services. Peri-domiciliary co-variables assessed included the presence of crops (yucca, corn, tomatoes, rice, banana, cocoa, and yam), the presence of vegetation (trees, shrubberies, and pastureland), the presence and husbandry purpose of domestic animals (canines, felines, pigs, equines, donkeys, mules, and birds), and the presence of wild animals, rodents and opossums, and household tick infestation, among others. Finally, family attitudes and practices, such as the use of long-sleeved and white shirts for working outdoors, floor fragmentation practices for agricultural purposes, rodent control, and canine hygiene (bathing and antiparasitic treatment), were analyzed.

### Statistical analysis

A descriptive analysis was conducted according to different outcomes; seroprevalent and seroincident cases, the relative and absolute frequencies for qualitative variables, and the median and interquartile ranges (IQR) for quantitative variables were estimated. The seroprevalence and seroincidence were estimated considering the number of cases in the numerator and, in the denominator, the number of total participants at baseline or one-year follow-up. The confidence intervals of seroprevalence and seroincidence were adjusted by random effect (hamlets).

A mixed-effects multinomial logistic regression model was conducted to estimate factors associated with different outcomes. The models were weighted by the inverse probability of the participants’ selection. All models included three levels: the individuals within households, households within hamlets, and hamlets (a random effect model using a variance component correlation matrix). The linearity assumption was confirmed by the inclusion of quantitative variables in models. A simple mixed-effects multinomial logistic regression model to bivariate analysis was conducted, and the variables included in the multivariable analysis were those with a p of value < 0.25. In addition, the stepwise method based on the purposeful selection of variables, including statistical and biological plausibility, was conducted in the multivariable analyses. The effect modification and confounding were evaluated, and the Bayesian Information Criteria (BIC) was used to select the best model that explained the outcomes. The Odds Ratio (OR) was estimated in the baseline study, and a complete descriptive analysis was conducted to evaluate the seroincident cases. All analyses were performed in SAS OnDemand^®^ software using procedures PROC FREQ, PROC UNIVARIATE, and PROC GLIMMIX.

## Results

### Participants

**Figure 3:**
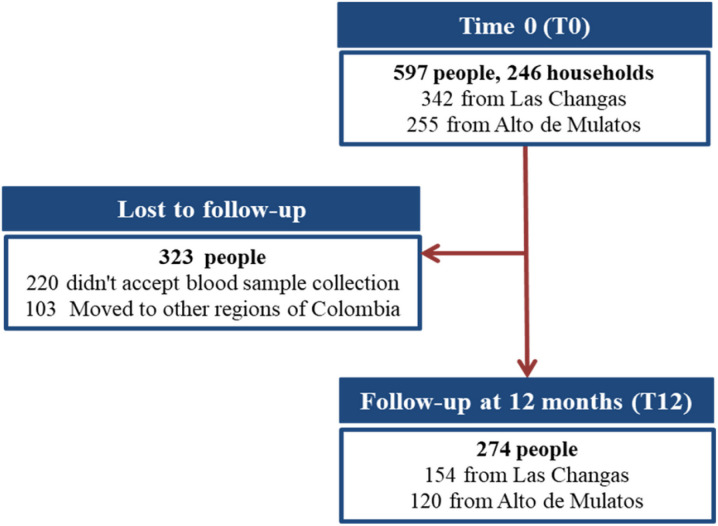
Flowchart of individuals enrolled in the study at T0 and T12.

At T0 (November 2015 to January 2016), 597 individuals residing in 246 households were included in the study (342 participants in Las Changas and 255 in Alto de Mulatos), surpassing the estimated number of households and achieving a participant sample coverage of 69.01%. At T12 (November 2016 to January 2017), 274 individuals who inhabited 152 households participated in the study. The reduced participation at T12 was attributed to some participants moving to other regions in search of work opportunities and the absence of consent from some participants for a second blood sample (Fig. 3).

### Epidemiological characterization of the seroprevalent cases against both Rickettsia and Leptospira

The seroprevalence against *Rickettsia* was 16.25% (97/597) [CI95% 13.06–20.03], against *Leptospira* was 18.43% (110/597) [CI95% 15.04–22.36], and concomitant seropositivity against *Rickettsia* and *Leptospira* was 9.38% (56/597) [CI 95% 6.08% –13.37%]. **S2 Table** shows the descriptive analysis for all the outcomes.

Of the concomitant seroprevalent cases, 78.57% (44/56) had positive serology against *L. interrogans* serogroups, and 21.42% (12/56) were seropositive to serogroups to other *Leptospira* species. On an individual level, 62.5% (35/56) of the cases were male, qith a median age of 35.02 years (IQR 20.46 to 52.68 years). Also, 66.07% (37/56) of the cases had had a history of fever, and 50% (28/56) worked in outdoor occupations.

Regarding household characteristics, 53.57% (30/56) of the cases lived in rural areas and predominantly resided in households with walls constructed partially or entirely of wood (94.64%; n = 53/56), soil floors (80.36% n = 45/56), and zinc roofs (60.71%; n = 34/56). Moreover, 48.21% (27/56) of the cases had aqueduct services, 14.29% (8/56) had sewerage services, and 50% (28/56) of the cases had designated garbage disposal areas. In terms of the presence of wild animals, 57.14% (32/56) and 82.14% (46/56) of the cases confirmed the presence of opossums and synanthropic rodents in peri-domicile areas, respectively. Likewise, 55.6% (31/56) of the seroprevalent cases reported having pigs, and 57.14% (32/56) had equids in their peri-domicile areas. Finally, according to the purpose of domestic animals, 33.93% (19/56) of the seroprevalent cases had breeding pigs, and 5.36% (3/56) had companion horses **(S2 Table)**.

### Bivariate and multivariate analysis for seroprevalent cases against both Rickettsia and Leptospira

The covariables included in the bivariate analysis (p < 0.25) are described in **S2 Table**. The bivariate models for seroprevalent cases against Rickettsia, against *Leptospira*, and concomitant seroprevalent cases are describe in Table 1.

The best multivariable model that explained the concomitant seroprevalent cases against *Rickettsia* and *Leptospira* adjusted by age, gender, and outdoor occupation, considered fever history, the presence of breeding pigs, deforestation practices, and yucca crops. Age and male gender were risk markers for seroprevalent cases against concomitant seroprevalent cases. Males had 2.06-fold more possibilities to be seroprevalent against *Rickettsia-Leptospira* (OR = 3.06; CI95%: 1.75–5.37) compared to females. The male gender was a confounding factor between the association of outdoor occupation and the concomitant seroprevalent cases (OR _outdoor occupation and gender_ = 12.52 CI 95% 8.99–17.74; OR _crude outdoor occupation_ = 4.38 CI 95%: 2.78–6.89; OR _outdoor occupation adjusted by gender_ = 2.84 CI 95%:1.68–4.78).

Likewise, for each additional year of age, the possibility of being a concomitant seroprevalent case increased by 2% (OR = 1.02; CI 95%: 1.007–1.03). Age was a confounding factor in the association between this outcome and having an outdoor occupation (OR _outdoor occupation and age_ = 1.04 CI 95% 1.034–1.05; OR _crude outdoor occupation_ = 4.38 CI 95%: 2.78–6.89; OR _outdoor occupation adjusted by age_ = 3.78 CI 95% 2.33–6.13). Additionally, at the individual level, the participants who reported fever history in the last year had a 1.71-fold possibility of being a concomitant seroprevalent case compared to the participants who didn’t report fever.

Finally, at the household level, the multivariate analysis showed that the factors associated with concomitant seroprevalent cases were breeding pigs (OR = 2.29; CI 95%: 1.36–3.88), the presence of peri-domiciliary yucca crops (O = 2.55; CI 95%: 1.16 – 5.62), and deforestation practices (O = 1.74; CI 95%: 1.06–2.87) (Table 1).

### Epidemiological characterization of the seroincident cases

The estimated seroincidence against *Rickettsia* was 4.22% (14/274) [CI95% 1.46–11.60], against *Leptospira* was 13.50% (37/274) [CI95% 9,41 – 19,00], and concomitant seroincidence against both *Rickettsia* and *Leptospira* was 1.09% (3/274) [CI 95%: 0.29–4.05%]. Table 2 provides an overview the characteristics of seroincident cases.

Two of the cases with concomitant seroincidence against *Rickettsia* and *Leptospira* had positive serology against *L. interrogans* serogroups. All the cases were female with a median age of 31.83 years (IQR 8.69–56.99), and the median time of residence in the area was 11 years (IQR 8–16). Only one of the cases reported a fever history during the last follow-up year.

At the household level, two of the concomitant seroincident cases against *Rickettsia* and *Leptospira* had households located in urban areas. Furthermore, all the cases resided in households constructed partially or entirely with soil floors, wooden walls, and zinc roofs. Two of the cases had households with garbage disposal areas and sewerage services. Regarding domestic animals, two of the cases owned equids, canines, and chickens, while one of the cases had pigs and turkeys. Based to the zootechnic purpose, two of the cases had companion canines, broilers, and laying hens. Similarly, one of the cases had dairy equines, companion equines, and charge equines. Two of the cases reported observing intra-domiciliary rodents, and one of the cases reported tick infestation and the presence of opossums in peri-domiciliary areas.

Finally, regarding attitudes and practices, two of the concomitant seroincident cases presented deforestation practices, used white clothes, and had the habit of removing ticks from the body. The description of the other variables for the outcome of concomitant seroincident cases is reported in Table 2.

## Discussion

This secondary data analysis showed the existence of susceptible individuals who presented concomitant seropositivity against *Rickettsia* and *Leptospira*. Furthermore, it identifies the individual and household factors associated with seroprevalent cases against *Rickettsia* and *Leptospira*. Additionally, this study assessed the isolated seroprevalence against *Rickettsia* and *Leptospira* and their associated factors. The results align to the baseline studies where multilevel factors such age, male gender, outdoor occupation, house construction material, peri-domiciliary domestic animals, peri-domiciliary opossums and rodents were associated with the seropositivity against these microorganisms (12–14).

The concomitant seroprevalent and seroincident findings provide evidence of individuals in Urabá, Colombia, who have been exposed to both microorganisms without developing overt clinical disease or being diagnosed. Our result carries important public health implications, as clinical case reports have indicated that individuals susceptible to concurrent infections by rickettsioses and leptospirosis may experience more severe clinical manifestations and potentially fatal outcomes(18, 22).

It’s worth noting that in Colombia, prior research has predominantly focused on the individual examination of seropositivity against either rickettsiae or leptospires, with limited reports of coinfections of rickettsioses and leptospirosis (17, 19, 23, 24). Therefore, this study is the first reported approximation in Colombia of the prevalence and the incidence of concomitant seropositivity against both *Rickettsia* and *Leptospira*.

In a review of the literature, a similar study conducted in four ecologically distinct regions in Perú reported a proportion of seroprevalent cases against both *Rickettsia* SFG and *Leptospira* at 1.8%, assuming that all exposure occurred within households (9). (25). In stark contrast, the present study unveiled a substantially higher seroprevalence against both microorganisms, registering at 9.38% (56/597) [CI 95% 6.08% – 13.37%], taking into account individual-level factors, household-level considerations (including domiciliary and peridomicile factors), as well as the knowledge and practices of the local population. This broader approach allows for a more nuanced understanding of the complex dynamics of exposure and susceptibility in the study region.

Having an outdoor occupation increases the risk exposure to the factors related to the transmission of these pathogenic agents, like water and soil contaminated with the urine of infected mammals, tick infestation, and contact with amplifier mammals (26, 27). In the original study, having an outdoor occupation was a factor that was associated with seroprevalence cases against *Rickettsia* (RP = 1.20; CI 95% 1.02–1.41), and against serogroups of *L. interrogans* (OR = 2.06; CI 95%:1.31–3.26) (12, 14). However, in the present secondary data analysis, when the variable outdoor occupation was adjusted by the other covariables in the multivariable model, there were no associations between the co-exposure seroprevalent cases against both *Rickettsia* and *Leptospira*.

According to the original study, males had greater possibility of being seroprevalent cases against *L. interrogans* and against *Rickettsia*, compared to females (12, 14). The results from this secondary data analysis showed that being male also was a risk marker for the concomitant seroprevalent cases against Rickettsia and *Leptospira*. Additionally, it was a confounding factor that overestimated the association between having an outdoor occupation and co-exposure seroprevalent cases. This finding can be rationalized by the fact that a significant proportion of male individuals who tested seroprevalent for both *Rickettsia* and *Leptospira* were employed in outdoor occupations, which consequently increased their exposure to these microorganisms.

The baseline study considered in this secondary analysis, along with other studies conducted in Chile and Cali, Colombia, have consistently demonstrated that age is a significant risk factor for seropositivity against *Rickettsia* and *Leptospira* serogroups (12, 28, 29). The results of this secondary data analysis further underscore the role of age as a risk marker associated with concomitant seroprevalent cases against *Rickettsia* and *Leptospira*, and that it was a confounding factor that overestimated the association between outdoor occupations and this outcome. Existing data had previously hinted at the fact that older individuals have a longer duration of exposure to *Ricketssia* and *Leptospira* agents, thereby increasing the possibility of being seropositive against both microorganisms (8, 23). Additionally, older individuals tend to be engaged in outdoor occupations, which further amplifies their exposure to factors associated with the transmission of these two pathogenic agents (30).

When exposures by rickettsiae and leptospires cause disease, it is manifested in a febrile syndrome associated with non-pathognomonic symptoms. This complicates diagnosis because rickettsioses and leptospirosis are part of the differential diagnosis of acute infectious febrile syndromes (1, 2). The results of the present secondary data analysis showed an association between fever history and concomitant seroprevalent cases against *Rickettsia* and *Leptospira*. These findings underline the importance for health professionals of considering that rickettsiae and leptospires are microorganisms that cause disease individually, sequentially, or in coinfection forms manifested like acute febrile syndromes.

At a household level, this study identified that the presence of breeding pigs increased the possibility of being a seroprevalent case against both *Rickettsia* and *Leptospira*. This finding aligns with results documented in the baseline study, which found that the presence of breeding pigs was associated with seroprevalent cases against *L. interrogans* (14). The association between breeding pigs and the concomitant seroprevalence to both agents can be elucidated by considering the role of infected or sick domestic pigs, which excrete leptospires in their urine, contaminating soils and water sources (31, 32) and the evidence of *Amblyomma cajennense* complex tick infestation in pigs, implicating them as vectors in the infection cycle of SFG rickettsiae (33, 34).

Yucca crops and deforestation practices were factors associated with seroprevalent cases against both *Rickettsia* and *Leptospira*. The presence of peri-domiciliary crops attracts synanthropic rodents and small wild mammals that search for food (35). Moreover, soil fragmentation and deforestation practices favor interaction among synanthropic rodents, wild mammals, domestic mammals used for agriculture activities, vectors, and humans, increasing the exposure to these infectious agents (36, 37).

Our study estimated a concomitant seroincidence of 1.09% [95% CI: 0.29–4.05%], below the 15% reported in Honduras for concomitant seroincident cases of both infectious agents (38). However, our findings highlight the need to recognize that individuals can acquire infections from both Rickettsia and Leptospira within a specific timeframe, due to Colombia has documented proportions ranging from 0.4–4.8% of rickettsiosis-leptospirosis coinfections in Urabá, Antioquia, and Villeta, Cundinamarca (17, 24). Therefore, strengthening the identification of sequential infections or coinfections involving rickettsioses and leptospirosis is essential for timely treatment and preventing fatal outcomes.

Among the individual characteristics of concomitant seroincident cases, it was observed that all cases were female. This observation may be attributed to the fact that a majority of males had relocated to other municipalities in search of employment by the 12-month follow-up period (13, 14). In addition, two of the concomitant seroincident cases worked in indoor occupations; hence, it is necessary to consider household conditions such as the presence of rodents and ticks, household building materials, contact with domestic animals, and women’s attitudes and practices as possible conditions that facilitated the presence of concomitant seroincident cases against *Rickettsia* and *Leptospira* (8, 39).

Different studies have proved that diverse *Leptospira* species can survive for months in wet soils rich in nitrates, iron, and copper, often more frequency than in water, suggesting that soil can can serve as a natural habitat and a potential environmental reservoir for *Leptospira* (40). Moreover, soil floors in indoor settings offer shelter to ectoparasites seeking refuge from adverse climatic conditions that hinder reproduction and increase mortality rates (41). Consequently, individuals residing in homes with soil floors, like the seroincident cases observed, may have increased contact with both rickettsiae and leptospires.

The base study identified canines and equines seropositive against *Rickettsia* and *Leptospira* (12). It also estimated that domestic seropositive animals and hunting canines were associated with the seropositivity against *Rickettsia* and *L. interrogans*, respectively (13, 14). This secondary data analysis described the presence of equines and canines in most concomitant seroincident cases against both *Rickettsia* and *Leptospira*, supporting the hypothesis that these domestic animals can be sentinels of infection for these pathogenic agents (42, 43). Additionally, domestic chickens, observed in concomitant seroincident cases, indirectly could relate to microorganism seropositivity due to attracting rodents, suggesting that waste management and coop maintenance could reduce exposure.

The evidence of rodents such as *Leptospira* reservoirs, the finding of rodents seropositive against SFG rickettsiaceae and the amplifiers host in common of these microorganism (44–47), provides a plausible explanation for the occurrence of simultaneous seroincident cases of *Rickettsia* and *Leptospira* in individuals living in areas with intra-domicile rodents and peri-domicile opossums.

Deforestation was a common practice in most of the concomitant seroincident cases, which indicates that educational strategies centered on the preservation of the vegetation and the adequate use of the soil should be prioritized. These strategies would help mitigate the impacts on the distribution of vector and animal species, thereby reducing alterations in the interactions among animals, vectors, and humans and minimizing contact with rickettsiae and leptospires (21, 48).

This study had some limitations. First, there was a potential selection bias due to the failure of the original study to reach the estimated sample coverage for individuals, resulting in a loss of follow-up of 45.89% of participants analyzed at T12 (12–14). Additionally, probable information biases were present, such as potential memory bias among participants during epidemiological surveys, and the generalization of attitudes and practices to other family members reported by the head of households in the household survey. Furthermore, certain variables of attitudes and practices related to *Leptospira*, such as drinking potable water, wearing footwear, avoiding swimming in stagnant water, and vaccinating pets, were not taken into account (49). Despite this limitation, the results of this research are still valid because the household survey implemented was designed for vector-transmitted diseases and evaluated many components that favor the presence of vectors and the conditions necessary for the transmission of *Rickettsia* and *Leptospira* agents. Finally, another potential information bias was related to the variation in sensitivity and specificity of serological assays used to determine antibodies titers against *Rickettsia* and *Leptospira*, depending on the timing of sample collection (50, 51). However, this study defined simultaneous exposure to both microorganisms using cut-off points of antibody titers clearly established in the evidence reported in other studies to improve the detection of seropositivity.

## Conclusions

The findings of this secondary data analysis demonstrated the potential concomitant exposure to bacteria of the genres *Rickettsia* and *Leptospira* in Urabá, Antioquia, Colombia, a region that presented rickettsioses outbreaks and which, during the period between 2015 and 2016, was one of the regions with the most notifications of leptospirosis cases in the epidemiologic surveillance system (52, 53)

It should be noted that the results of this study found that individuals can be exposed to both microorganisms, evidenced by concomitant seroprevalent and seroincident cases against *Rickettsia* and *Leptospira*.

Rickettsiae and leptospires are microorganisms with a complex infection cycle that involve numerous factors, which can be addressed from an eco-epidemiological perspective. Our results showed that specific factors can plausibly facilitate the transmission of both microorganisms in humans: individual factors like age and male gender, outdoor occupation and fever history; household factors such as building materials, the presence of domestic, synanthropic, wild animals; and human attitudes and practices.

Awareness of concomitant seroprevalent and seroincident cases against *Rickettsia* and *Leptospira*, and the ability to identify their associated factors, will allow us to consider the inclusion of rickettsioses into the epidemiologic surveillance system in Colombia. It will also allow the strengthening and creation of implementation strategies to improve the diagnosis and timely treatment of possible coinfections and, finally, the creation of measures centered on improving household conditions, sanitary measures, access to public services, animal care, control of rodents and ectoparasite infestations, and raising awareness of deforestation and soil fragmentation practices.

## Figures and Tables

**Table 1:** Multinomial mixed methods multivariate analysis for seroprevalent cases against **Rickettsia, Leptospira** and for both

Variables	Total. n = 597	Seronegative cases. n = 334	Seroprevalent cases against *Rickettsia* n = 97	OR Adjusted^a^ [CI 95%]	Seroprevalent cases against *Leptospira* n = 110	OR Adjusted^a^ [CI 95%]	Concomitant seroprevalent cases. n = 56	OR Adjusted^a^ [CI 95%]
Individual Level	n (%)	n (%)	n (%)		n (%)		n (%)	
Age (years)- Median (IQR)	29.7 (15.29–46.11)	27.05 (14.06–43.70)	38.90 (17.49–55.43)	1.02 [1.009–1.03]	28.57 (17.17–45.70)	1.005 [0.99–1.01]	35.01 (20.46–52.69)	1.02 [1.007–1.03]
Gender (males)	233 (39.03)	106 (31.74)	40 (41.24)	1.42 [0.92–2.19]	52 (47.27)	1.55 [1.03–2.32]	35 (62.5)	3.06 [1.75–5.37]
Outdoor occupation	177 (29.65)	69 (20.66)	35 (36.08)	1.26 [0.78–2.04]	43 (39.09)	1.95 [1.24–3.06]	30 (53.57)	1.59 [0.86–2.91]
Fever history in the last year	350 (58.63)	189 (56.59)	-	-	-	-	37 (66.07)	1.71 [1.06–2.77]
**Household level**								
Block wall	175 (29.31)	108 (32.34)	22 (22.68)	0.60 [0.40–0.91]	-	-	-	-
Zinc roof	407 (68.17)	242 (72.46)	–	–	69 (62.73)	0.67 [0.47–0.97]	–	–
Equids	301 (50.42)	153 (45.81)	61 (62.89)	1.62 [1.12–2.35]	–	–	–	–
Breeding pigs	122 (20.44)	60 (17.96)	–	–	–	–	19 (33.93)	2.29 [1.36–3.88]
Companion chickens			–	–	10 (9.09)	2.33 [1.18–4.56]	–	–
Intra-domicile rodents	481 (80.57)	264 (79.04)	–	–	95 (86.36)	1.80 [1.11–2.90]	–	–
Peri-domicile opossums	288 (48.81)	140 (42.68)	56 (58.33)	1.56 [1.08–2.26]	60 (54.55)	1.44 [1.01–2.04]	–	–
Yucca	36 (6.03)	18 (3.02)	–	–	–	–	7 (1.17)	2.5 [1.16–5.62]
Deforestation practices	378 (63.32)	199 (33.33)	–	–	–	–	40 (6.70)	1.74 [1.06–2.87]

**Table 2:** Descriptive analysis of the covariables for seroincident cases

Variables	Total n = 274	Seronegative cases n = 220	Concomitant seroincident cases against *Rickettsia-Leptospira* n = 3	Seroincident cases against *Rickettsia* n = 14	Seroincident cases against *Leptospira* n = 37
	n (%)	n (%)	n (%)	n (%)	n (%)

Individual level					

Age (years)- Median (IQR)	34.78 (18.20-52.02)	33.70 (18.01–50.13)	31.83 (8.69–56.99)	44.46 (18.61–54)	41.44 (21.08–53.68)

Sex (males)	83 (30.29)	69 (31.36)	0	3 (21.43)	11 (29.73)

Ethnicity (Amerindians or afro-descendant)	17 (6.23)	15 (6.82)	0	0	2 (5.56)

Outdoor occupation	79 (28.83)	61 (27.73)	1 (33.33)	4 (28.57)	13 (35.14)

Time of residence in the area (years)- Median (IQR)	13 (7–22)	13 (7–23)	11 (8–16)	11 (5–16)	14 (8–21)

Fever history in the last year	14 (5.51)	11 (5.0)	1 (33.33)	2 (14.29)	0

**Household level**					

Household location (urban)	127 (46.35)	104 (47.27)	2 (66.67)	7 (50)	14 (37.84)

Proximity among houses	64 (23.36)	51 (23.18)	1 (33.33)	5 (35.71)	7 (18.92)
Widely dispersed	55 (20.07)	41 (18.64)	1 (33.33)	2 (14.29)	11 (29.73)
Dispersed		62 (28.18)	1 (33.33)	2 (14.29)	9 (24.32)
concentrated	74 (27.01)	66 (30.0)	0	5 (35.71)	10 (27.03)
widely concentrated	81 (29.56)				

Soil floor	187 (68.25)	144 (65.45)	3 (100)	11 (78.57)	29 (78.38)

Concrete floor	112 (40.88)	97 (44.09)	1 (33.33)	5 (35.71)	9 (24.32)

Tile floor	16 (5.84)	13 (5.91)	0	1 (7.14)	2 (5.41)

Wood floor	23 (8.39)	22 (10)	1 (33.33)	0	0

Concrete wall	83 (30.29)	73 (33.18)	0	4 (28.57)	6 (16.22)

Wood wall	246 (89.78)	196 (89.09)	3 (100)	13 (92.86)	34 (91.89)

Bamboo wall	2 (0.73)	1 (0.45)	0	0	1 (2.70)

Bahareque wall	2 (0.73)	1 (0.45)	0	0	1 (2.70)

Vegetal roof	136 (49.64)	106 (48.18)	1 (33.33)	8 (57.14)	21 (56.76)

Zinc roof	181 (66.06)	144 (65.45)	3 (100)	11 (78.57)	23 (62.16)

**Variables**	**Total n = 274**	**Seronegative cases n = 220**	**Concomitant seroincident cases against RickettsiaLeptospira n = 3**	**Seroincident cases against Rickettsia n = 14**	**Seroincident cases against Leptospira n = 37**

	**n (%)**	**n (%)**	**n (%)**	**n (%)**	**n (%)**

Tile roof	15 (5.47)	15 (6.82)	0	0	0

Wood roof	24 (8.76)	20 (9.09)	0	0	4 (10.81)

Garbage disposure area	142 (51.82)	119 (54.09)	2 (66.67)	7 (50)	14 (37.84)

Presence of septic tank	30 (10.95)	19 (8.64)	0	4 (28.57)	7 (18.92)

Presence of toilet	130 (47.45)	106 (48.18)	1 (33.33)	7 (50)	16 (43.24)

Aqueduct service	146 (53.28)	119 (54.09)	2 (66.67)	10 (71.43)	15 (40.54)

Sewerage service	34 (12.41)	30 (13.64)	1 (33.33)	0	3 (8.11)

Light service	270 (98.54)	219 (99.55)	3 (100)	13 (92.86)	35 (94.59)

**Presence of peri-domicile domestic animals**					

Canines	161 (58.76)	129 (58.64)	2 (66.67)	6 (42.86)	24 (64.86)

Felines	170 (62.04)	134 (60.91)	3 (100)	10 (71.43)	23 (62.16)

Equids	147 (53.65)	117 (53.18)	2 (66.67)	9 (64.29)	19 (12.93)

Horses	81 (29.56)	64 (29.09)	2 (66.67)	5 (35.71)	10 (27.03)

Mules	34 (12.41)	30 (13.64)	0	2 (14.29)	2 (5.41)

Donkeys	89 (32.48)	71 (32.27)	2 (66.67)	3 (21.43)	13 (35.14)

Pigs	135 (49.27)	110 (50)	1 (33.33)	5 (35.71)	19 (51.35)

Chickens	197 (71.90)	164 (74.55)	2 (66.67)	7 (50)	24 (64.86)

Turkeys	66 (24.09)	52 (23.64)	1 (33.33)	3 (21.43)	10 (27.03)

**Zootechnie purpose of domestic animals**					

Companion canines	154 (56.20)	124 (56.36)	2 (66.67)	6 (42.86)	22 (59.46)

Hunting canines	10 (3.65)	7 (3.18)	0	0	3 (8.11)

Meat pigs	116 (42.34)	94 (42.73)	1 (33.33)	5 (35.71)	16 (43.24)

Breeding pigs	64 (23.36)	48 (21.82)	1 (33.33)	4 (28.57)	11 (29.73)

Companion pigs	4 (146)	3 (1.36)	0	0	1 (2.70)

**Variables**	**Total n = 274**	**Seronegative cases n = 220**	**Concomitant seroincident cases against RickettsiaLeptospira n = 3**	**Seroincident cases against Rickettsia n = 14**	**Seroincident cases against Leptospira n = 37**

	**n (%)**	**n (%)**	**n (%)**	**n (%)**	**n (%)**

Meat chickens	110 (40.15)	91 (41.36)	2 (66.67)	5 (35.71)	12 (32.43)

Eggs chickens	157 (57.30)	131 (59.55)	2 (66.67)	5 (35.71)	19 (51.35)

Companion chickens	11 (4.01)	8 (3.64)	0	0	3 (8.11)

Meat turkeys	55 (20.07)	44 (20)	1 (33.33)	3 (21.43)	7 (18.92)

Eggs turkeys	48 (17.52)	37 (16.82)	1 (33.33)	3 (21.43)	7 (18.92)

Companion turkeys	3 (1.09)	2 (0.91)	0	0	1 (2.70)

Pack horses	57 (20.80)	43 (19.55)	1 (33.33)	4 (28.57)	9 (24.32)

Dairy horses	30 (10.95)	26 (11.82)	1 (33.33)	1 7.14)	2 (5.41)

Companion horses	8 (2.92)	6 (2.73)	1 (33.33)	1 (7.14)	0

Pack donkeys	88 (32.12)	70 (31.82)	2 (66.67)	3 (21.43)	13 (35.14)

Companion donkeys	1 (0.36)	1 (0.45)	0	0	0

Pack mules	33 (12.04)	29 (13.18)	0	2 (14.29)	2 (5.41)

Dairy mules	5 (1.82)	5 (2.27)	0	0	0

**Presence of ectoparasites, synanthropic and wild mammals**					

Intra-domicile rodents	212 (77.37)	166 (75.45)	2 (66.67)	12 (85.71)	32 (86.49)

Peri-domicile opossums	151 (55.31)	127 (57.73)	1 (33.33)	7 (50)	16 (44.44)

Pero-domicile wild mammals	43 (16.41)	34 (16.19)	1 (33.33)	2 (14.29)	6 (17.14)

Intra-domicile tick infestation	85 (31.02)	71 (32.27)	1 (33.33)	4 (28.57)	9 (24.32)

**Crops and vegetation in peri-domicile**					

Corn	4 (146)	2 (0.91)	0	0	2 (5.41)

Yucca	21 (7.66)	14 (6.36)	0	1 (7.14)	6 (16.22)

Yam	9 (3.28)	6 (2.73)	0	0	3 (3.28)

Tomato	5 (1.82)	4 (1.82)	0	0	1 (2.70)

Cocoa	12 (4.38)	9 (4.09)	0	1 (7.14)	2 (5.41)

**Variables**	**Total n = 274**	**Seronegative cases n = 220**	**Concomitant seroincident cases against RickettsiaLeptospira n = 3**	**Seroincident cases against Rickettsia n = 14**	**Seroincident cases against Leptospira n = 37**

	**n (%)**	**n (%)**	**n (%)**	**n (%)**	**n (%)**

Banana	31 (11.31)	24 (10.91)	1 (33.33)	3 (21.43)	3 (8.11)

Trees	254 (92.70)	203 (92.27)	2 (66.67)	13 (92.86)	36 (97.30)

Shrubbery	254 (92.70)	206 (93.64)	2 (66.67)	13 (92.86)	33 (89.19)

Pastureland	146 (53.28)	118 (53.64)	2 (66.67)	5 (35.71)	21 (56.76)

**Attitudes and practices**					

Deforestation practices	170 (62.04)	135 (61.36)	2 (66.67)	9 (64.29)	24 (64.86)

Use of long-sleeved shirt	67 (24.45)	52 (23.64)	0	5 (35.71)	10 (27.03)

Use of white clothes	198 (72.26)	161 (73.18)	2 (66.67)	10 (71.43)	25 (67.57)

Canine cleaning	50 (18.25)	39 (17.73)	1 (33.33)	3 (21.43)	7 (18.92)

Tick removal	117 (42.07)	88 (40)	2 (66.67)	10 (71.43)	17 (45.95)

## Data Availability

The datasets generated and/or analyzed during the current study are not publicly available due to this study is a secondary data analysis from a prior study but are available from the corresponding author on reasonable request.

## Abbreviations

BIC: bayesian Information Criteria
CI: confidence interval
IFA: indirect immunofluorescence assay
IgG: immunoglobulin G
IQR: Interquartile range
MAT: microscopic agglutination testing
OR: Odds Ratio
SFG: Spotterd fever group

## References

[1] Blanton LS. The Rickettsioses. Infect Dis Clin N Am. 2019;33(1):213–29. 10.1016/j.idc.2018.10.010 PMC6364315 30712763

[2] Haake DA, Levett PN. Leptospirosis in Humans. In: Adler B,Leptospira and Leptospirosis [Internet]., Berlin. Heidelberg: Springer Berlin Heidelberg; 2015 [cited 2023 Sep 19]. p. 65–97. (Current Topics in Microbiology and Immunology; vol. 387). Available from: 10.1007/978-3-662-45059-8_5.

[3] Thibeaux R, Girault D, Bierque E, Soupé-Gilbert ME, Rettinger A, Douyère A, Biodiversity of Environmental Leptospira: Improving Identification and Revisiting the Diagnosis. Front Microbiol. 2018;9:816. 29765361 10.3389/fmicb.2018.00816 PMC5938396

[4] Chin VK, Basir R, Nordin SA, Abdullah M, Sekawi Z. Pathology and Host Immune Evasion During Human Leptospirosis: a Review. Int Microbiol. 2020;23(2):127–36. 30875033 10.1007/s10123-019-00067-3

[5] Rydkina E, Turpin LC, Sahni SK. *Rickettsia rickettsii* Infection of Human Macrovascular and Microvascular Endothelial Cells Reveals Activation of Both Common and Cell Type-Specific Host Response Mechanisms. Infect Immun. 2010;78(6):2599–606. 20385756 10.1128/IAI.01335-09 PMC2876542

[6] Parola P, Paddock CD, Socolovschi C, Labruna MB, Mediannikov O, Kernif T, Update on Tick-Borne Rickettsioses around the World: a Geographic Approach. Clin Microbiol Rev. 2013;26(4):657–702. 24092850 10.1128/CMR.00032-13 PMC3811236

[7] Chikeka I, Dumler JS. Neglected bacterial zoonoses. Clin Microbiol Infect. 2015;21(5):404–15. 25964152 10.1016/j.cmi.2015.04.022 PMC4466158

[8] Padmanabha H, Hidalgo M, Valbuena G, Castaneda E, Galeano A, Puerta H, Geographic Variation in Risk Factors for SFG Rickettsial and Leptospiral Exposure in Colombia. Vector-Borne and Zoonotic Diseases. 2009;9(5):483–90. 18973451 10.1089/vbz.2008.0092

[9] Salmon-Mulanovich G, Simons MP, Flores-Mendoza C, Loyola S, Silva M, Kasper M, Seroprevalence and Risk Factors for Rickettsia and Leptospira Infection in Four Ecologically Distinct Regions of Peru. Am J Trop Med Hyg. 2019;100(6):1391–400. 30938281 10.4269/ajtmh.18-0029 PMC6553916

[10] Tomassone L, Berriatua E, De Sousa R, Duscher GG, Mihalca AD, Silaghi C, Neglected vector-borne zoonoses in Europe: Into the wild. Vet Parasitol. 2018;251:17–26. 29426471 10.1016/j.vetpar.2017.12.018

[11] Cámara de Comercio de Urabá. Perfil socioeconómico de la subregión del Urabá. 2021.

[12] Quintero VJC, Paternina TLE, Uribe YA, Muskus C, Gil HM J, Eco-epidemiological analysis of rickettsial seropositivity in rural areas of Colombia: A multilevel approach. Foley J, editor. PLoS Negl Trop Dis. 2017;11(9):e0005892. 28922404 10.1371/journal.pntd.0005892 PMC5619838

[13] Quintero Vélez JC, Aguirre-Acevedo DC, Rodas JD, Arboleda M, Troyo A, Vega Aguilar F Epidemiological characterization of incident cases of Rickettsia infection in rural areas of Urabá region, Colombia. Nieto NC, editor. PLoS Negl Trop Dis. 2018;12(10):e0006911. 30379820 10.1371/journal.pntd.0006911 PMC6242695

[14] Quintero-Vélez JC, Rodas JD, Rojas CA, Ko AI, Wunder EA. Leptospira Infection in Rural Areas of Urabá Region, Colombia: A Prospective Study. Am J Trop Med Hyg. 2022;107(6):1267–77. 36375452 10.4269/ajtmh.21-1103 PMC9768283

[15] Instituto Nacional de Salud. Protocolo de vigilancia de leptospirosis.

[16] Cortés JA, Romero Moreno LF, Aguirre León CA, Pinzón Lozano L, Cuervo SI. Enfoque clínico del síndrome febril agudo en Colombia. Infect [Internet]. 2017 Jan 20 [cited 2023 Sep 20];21(1). Available from: https://revistainfectio.org/P_OJS/index.php/infectio/article/view/640.

[17] Arroyave E, Londoño AF, Quintero JC, Agudelo-Florez P, Arboleda M, Díaz FJ Etiología y caracterización epidemiológica del síndrome febril no malárico en tres municipios del Urabá antioqueño, Colombia. biomedica [Internet]. 2012 Sep 4 [cited 2023 Sep 20];33(0). Available from: http://www.revistabiomedica.org/index.php/biomedica/article/view/734. 24652254

[18] Ramírez-García R, Quintero JC, Rosado AP, Arboleda M, González VA, Agudelo-Flórez P. Leptospirosis y rickettsiosis, reto diagnóstico para el síndrome febril en zonas endémicas. biomedica. 2021;41(2):208–17. 34214261 10.7705/biomedica.5598 PMC8372841

[19] Pérez-García J, Agudelo-Flórez P, Parra-Henao GJ, Ochoa JE, Arboleda M. Incidencia y subregistro de casos de leptospirosis diagnosticados con tres métodos diferentes en Urabá. Colombia Biomed. 2019;39:150–62. 10.7705/biomedica.v39i0.4577 31529857

[20] Londoño AF, Acevedo-Gutiérrez LY, Marín D, Contreras V, Díaz FJ, Valbuena G, Human prevalence of the spotted fever group (SFG) rickettsiae in endemic zones of Northwestern Colombia. Ticks and Tick-borne Diseases. 2017;8(4):477–82. 28223058 10.1016/j.ttbdis.2017.02.006

[21] Portillo A, De Sousa R, Santibáñez S, Duarte A, Edouard S, Fonseca IP, Guidelines for the Detection of *Rickettsia* spp. Vector-Borne and Zoonotic Diseases. 2017;17(1):23–32. 28055574 10.1089/vbz.2016.1966

[22] Harada Y, Hayashi M. Severe leptospirosis in a patient with positive serological test for spotted fever rickettsiosis. BMJ Case Rep. 2019;12(1):bcr–2018. 10.1136/bcr-2018-226514 PMC6340506 30635303

[23] Ferro BE, Rodríguez AL, Pérez M, Travi BL. Seroprevalencia de infección por Leptospira en habitantes de barrios periféricos de Cali. biomedica. 2006;26(2):250. 16925097

[24] Valbuena E, Faccini-Martínez ÁA, Barreto C, Palomar AM, Polo-Terán LJ, Imbacuán-Pantoja WO, Epidemiology of Spotted Fever Group Rickettsioses and Acute Undifferentiated Febrile Illness in Villeta, Colombia. Am J Trop Med Hyg. 2017;97(3):782–8. 28722568 10.4269/ajtmh.16-0442 PMC5590559

[25] Salmon-Mulanovich G, Simons MP, Flores-Mendoza C, Loyola S, Silva M, Kasper M, Seroprevalence and risk factors for rickettsia and leptospira infection in Four Ecologically Distinct Regions of Peru. Am J Trop Med Hyg. 2019;100(6):1391–400. 30938281 10.4269/ajtmh.18-0029 PMC6553916

[26] El-Tras WF, Bruce M, Holt HR, Eltholth MM, Merien F. Update on the status of leptospirosis in New Zealand. Acta Trop. 2018;188:161–7. 30165070 10.1016/j.actatropica.2018.08.021

[27] Noden BH, Tshavuka FI, Van Der Colf BE, Chipare I, Wilkinson R. Exposure and Risk Factors to Coxiella burnetii, Spotted Fever Group and Typhus Group Rickettsiae, and Bartonella henselae among Volunteer Blood Donors in Namibia. Yu X jie, editor. PLoS ONE. 2014;9(9):e108674. 25259959 10.1371/journal.pone.0108674 PMC4178180

[28] Weitzel T, Acosta-Jamett G, Jiang J, Martínez-Valdebenito C, Farris CM, Richards AL, Human seroepidemiology of Rickettsia and Orientia species in Chile – A cross-sectional study in five regions. Ticks and Tick-borne Diseases. 2020;11(6):101503. 32993924 10.1016/j.ttbdis.2020.101503

[29] Escandón-Vargas K, Osorio L, Astudillo-Hernández M. Seroprevalence and factors associated with Leptospira infection in an urban district of Cali, Colombia. Cad Saúde Pública [Internet]. 2017 [cited 2023 Sep 22];33(5). Available from: http://www.scielo.br/scielo.php?script=sci_arttext&pid=S0102-311X2017000505004&lng=en&tlng=en. 10.1590/0102-311X00039216 28614448

[30] Lau CL, Watson CH, Lowry JH, David MC, Craig SB, Wynwood SJ Human Leptospirosis Infection in Fiji: An Eco-epidemiological Approach to Identifying Risk Factors and Environmental Drivers for Transmission. Picardeau M, editor. PLoS Negl Trop Dis. 2016;10(1):e0004405. 26820752 10.1371/journal.pntd.0004405 PMC4731082

[31] Kurilung A, Chanchaithong P, Lugsomya K, Niyomtham W, Wuthiekanun V, Prapasarakul N. Molecular detection and isolation of pathogenic Leptospira from asymptomatic humans, domestic animals and water sources in Nan province, a rural area of Thailand. Res Vet Sci. 2017;115:146–54. 28384550 10.1016/j.rvsc.2017.03.017

[32] Fernandes JJ, Araújo Júnior JP, Malossi CD, Ullmann LS, Da Costa DF, Silva MLCR, High frequency of seropositive and carriers of Leptospira spp. in pigs in the semiarid region of northeastern Brazil. Trop Anim Health Prod. 2020;52(4):2055–61. 32026195 10.1007/s11250-020-02203-y

[33] Osava CF, Ramos VDN, Rodrigues AC, Dos Reis Neto HV, Martins MM, Pascoal JO, Amblyomma sculptum (Amblyomma cajennense complex) tick population maintained solely by domestic pigs. Veterinary Parasitology: Regional Studies and Reports. 2016;6:9–13. 10.1016/j.vprsr.2016.11.002 31014529

[34] Bermúdez SE, Eremeeva ME, Karpathy SE, Samudio F, Zambrano ML, Zaldivar Y, Detection and Identification of Rickettsial Agents in Ticks From Domestic Mammals in Eastern Panama. me. 2009;46(4):856–61. 10.1603/033.046.0417 19645289

[35] Mkenda PA, Ndakidemi PA, Mbega E, Stevenson PC, Arnold SEJ, Gurr GM, Multiple ecosystem services from field margin vegetation for ecological sustainability in agriculture: scientific evidence and knowledge gaps. PeerJ. 2019;7:e8091. 31799074 10.7717/peerj.8091 PMC6885351

[36] Morand S, Blasdell K, Bordes F, Buchy P, Carcy B, Chaisiri K, Changing landscapes of Southeast Asia and rodent-borne diseases: decreased diversity but increased transmission risks. Ecol Appl. 2019;29(4):e01886. 30986339 10.1002/eap.1886

[37] Scinachi CA, Takeda GACG, Mucci LF, Pinter A. Association of the occurrence of Brazilian spotted fever and Atlantic rain forest fragmentation in the São Paulo metropolitan region, Brazil. Acta Trop. 2017;166:225–33. 27880877 10.1016/j.actatropica.2016.11.025

[38] Chao CC, Zhang Z, Belinskaya T, Chen HW, Ching WM. Leptospirosis and Rickettsial Diseases Sero-Conversion Surveillance Among U.S. Military Personnel in Honduras. Mil Med. 2022;187(7–8):802–7. 33861353 10.1093/milmed/usab120

[39] Berrian AM, Martínez-López B, Quan V, Conrad PA, Van Rooyen J, Simpson GJG, Risk factors for bacterial zoonotic pathogens in acutely febrile patients in Mpumalanga Province, South Africa. Zoonoses Public Health. 2019;66(5):458–69. 30859717 10.1111/zph.12577

[40] Bierque E, Thibeaux R, Girault D, Soupé-Gilbert ME, Goarant C. A systematic review of Leptospira in water and soil environments. Dellagostin OA. editor PLoS ONE. 2020;15(1):e0227055. 31986154 10.1371/journal.pone.0227055 PMC6984726

[41] Burtis JC, Yavitt JB, Fahey TJ, Ostfeld RS. Ticks as Soil-Dwelling Arthropods: An Intersection Between Disease and Soil Ecology. Ginsberg H, editor. Journal of Medical Entomology. 2019;56(6):1555–64. 31318035 10.1093/jme/tjz116

[42] Bowser N, Anderson N. Dogs (Canis familiaris) as Sentinels for Human Infectious Disease and Application to Canadian Populations: A Systematic Review. Veterinary Sci. 2018;5(4):83. 10.3390/vetsci5040083 PMC6313866 30248931

[43] Souza CE, Camargo LB, Pinter A, Donalisio MR. High Seroprevalence for Rickettsia rickettsii in Equines Suggests Risk of Human Infection in Silent Areas for the Brazilian Spotted Fever. Yu X jie, editor. PLoS ONE. 2016;11(4):e0153303. 27064788 10.1371/journal.pone.0153303 PMC4827800

[44] Boey K, Shiokawa K, Rajeev S. Leptospira infection in rats: A literature review of global prevalence and distribution. Day NP, editor. PLoS Negl Trop Dis. 2019;13(8):e0007499. 31398190 10.1371/journal.pntd.0007499 PMC6688788

[45] Lopes MG, Muñoz-Leal S, De Lima JTR, Fournier GFDSR, Acosta IDCL, Martins TF, Ticks, rickettsial and erlichial infection in small mammals from Atlantic forest remnants in northeastern Brazil. Int J Parasitology: Parasites Wildl. 2018;7(3):380–5. 10.1016/j.ijppaw.2018.10.001 PMC6199183 30370217

[46] Quintero JC, Londoño AF, Díaz FJ, Agudelo-Flórez P, Arboleda M, Rodas JD. Ecoepidemiología de la infección por rickettsias en roedores, ectoparásitos y humanos en el noroeste de Antioquia, Colombia. biomedica [Internet]. 2012 Sep 4 [cited 2023 Sep 22];33(0). Available from: http://www.revistabiomedica.org/index.php/biomedica/article/view/735. 24652248

[47] Samrot AV, Sean TC, Bhavya KS, Sahithya CS, Chan-drasekaran S, Palanisamy R, Leptospiral Infection, Pathogenesis and Its Diagnosis—A Review. Pathogens. 2021;10(2):145. 33535649 10.3390/pathogens10020145 PMC7912936

[48] Felzemburgh RDM, Ribeiro GS, Costa F, Reis RB, Hagan JE, Melendez AXTO Prospective Study of Leptospirosis Transmission in an Urban Slum Community: Role of Poor Environment in Repeated Exposures to the Leptospira Agent. Haake DA, editor. PLoS Negl Trop Dis. 2014;8(5):e2927. 24875389 10.1371/journal.pntd.0002927 PMC4038618

[49] Center for Disease. Control and Prevention. Leptospirosis. 2015.

[50] Alugubelly N, Stokes JV, Cross CE, Ross AML, Crawford AE, Fiihr GF, Beyond the IFA: Revisiting the ELISA as a More Sensitive, Objective, and Quantitative Evaluation of Spotted Fever Group Rickettsia Exposure. Pathogens. 2021;10(2):88. 33498380 10.3390/pathogens10020088 PMC7909427

[51] Marquez A, Djelouadji Z, Lattard V, Kodjo A. Overview of laboratory methods to diagnose Leptospirosis and to identify and to type leptospires. Int Microbiol Official J Span Soc Microbiol. 2017;(20):184–93. 10.2436/20.1501.01.302 29529330

[52] Instituto Nacional de Salud. Boletin epidemiológico semana 52 año 2015. 2015.

[53] Instituto Nacional de Salud. Boletín epidemiológico semana 52 año 2016.

